# Supplementary material for: Dynamic regulation of chromatin accessibility by pluripotency transcription factors across the cell cycle
Source: eLife. 2019 Dec 3;8:e50087. doi: 10.7554/eLife.50087 (PMC6890464; doi:10.7554/eLife.50087)
Supplement: Transparent reporting form [file elife-50087-transrepform.docx]

***eLife’s* transparent reporting form**

We encourage authors to provide detailed information *within their submission* to facilitate the interpretation and replication of experiments. Authors can upload supporting documentation to indicate the use of appropriate reporting guidelines for health-related research (see [EQUATOR Network](http://www.equator-network.org/%20)), life science research (see the [BioSharing Information Resource](https://biosharing.org/)), or the [ARRIVE guidelines](http://www.plosbiology.org/article/info:doi/10.1371/journal.pbio.1000412) for reporting work involving animal research. Where applicable, authors should refer to any relevant reporting standards documents in this form.

If you have any questions, please consult our Journal Policies and/or contact us: [editorial@elifesciences.org](mailto:editorial@elifesciences.org).

**Sample-size estimation**

- You should state whether an appropriate sample size was computed when the study was being designed
- You should state the statistical method of sample size computation and any required assumptions
- If no explicit power analysis was used, you should describe how you decided what sample (replicate) size (number) to use

Please outline where this information can be found within the submission (e.g., sections or figure legends), or explain why this information doesn’t apply to your submission:

Two to three replicates of sequencing experiments were based on recommendations from the ENCODE project. Other experiments were biologically replicated 3-5 times, without explicit power calculations. Figure 1 – figure supplement 1B contains one condition with two technical replicates and Figure 4B-C are based on one replicate, but in these experiments we measured multiple cells individually (source data available).

**Replicates**

- You should report how often each experiment was performed
- You should include a definition of biological versus technical replication
- The data obtained should be provided and sufficient information should be provided to indicate the number of independent biological and/or technical replicates
- If you encountered any outliers, you should describe how these were handled
- Criteria for exclusion/inclusion of data should be clearly stated
- High-throughput sequence data should be uploaded before submission, with a private link for reviewers provided (these are available from both GEO and ArrayExpress)

Please outline where this information can be found within the submission (e.g., sections or figure legends), or explain why this information doesn’t apply to your submission:

Replicate information is either in the methods or in the Figure legends. Biological replicates indicate separate cell seeding days (all experiments) or separate passages of the cell line(s) (RT-qPCR experiments). Technical replicates indicate different number of cells seeded at the beginning of the experiment. We did not exclude any samples or outliers. We have uploaded high-throughput sequencing data to the GEO database (GSE134680).

**Statistical reporting**

- Statistical analysis methods should be described and justified
- Raw data should be presented in figures whenever informative to do so (typically when N per group is less than 10)
- For each experiment, you should identify the statistical tests used, exact values of N, definitions of center, methods of multiple test correction, and dispersion and precision measures (e.g., mean, median, SD, SEM, confidence intervals; and, for the major substantive results, a measure of effect size (e.g., Pearson's r, Cohen's d)
- Report exact p-values wherever possible alongside the summary statistics and 95% confidence intervals. These should be reported for all key questions and not only when the p-value is less than 0.05.

Please outline where this information can be found within the submission (e.g., sections or figure legends), or explain why this information doesn’t apply to your submission:

For the determination of statistically significant changes in chromatin accessibility we use the well-documented and established limma/voom algorithm using a false discovery rate of <5%, as described in the Materials and methods and main text. Motif enrichment statistics are determined using the well-established HOMER algorithm, as described in the Materials and methods. Other statistical methods are described and justified in the Materials and methods section and the tests indicated for each figure in the Figure or Table legends. We have reported all p-values in figures or as separate tables when many comparisons were made.

(For large datasets, or papers with a very large number of statistical tests, you may upload a single table file with tests, Ns, etc., with reference to sections in the manuscript.)

**Group allocation**

- Indicate how samples were allocated into experimental groups (in the case of clinical studies, please specify allocation to treatment method); if randomization was used, please also state if restricted randomization was applied
- Indicate if masking was used during group allocation, data collection and/or data analysis

Please outline where this information can be found within the submission (e.g., sections or figure legends), or explain why this information doesn’t apply to your submission:

Groups were always done in a paired setup, i.e. cells were plated on the same day in different plates/wells and then treated differently, or sorted based on cell cycle phase from the same initial population.

**Additional data files (“source data”)**

- We encourage you to upload relevant additional data files, such as numerical data that are represented as a graph in a figure, or as a summary table
- Where provided, these should be in the most useful format, and they can be uploaded as “Source data” files linked to a main figure or table
- Include model definition files including the full list of parameters used
- Include code used for data analysis (e.g., R, MatLab)
- Avoid stating that data files are “available upon request”

Please indicate the figures or tables for which source data files have been provided:

Raw and processed sequencing data have been submitted to GEO under the accession number GSE134680. Source data for Figure 4B-C have been deposited.
